# Correction: Interactions of L-3,5,3'-Triiodothyronine, Allopregnanolone, and Ivermectin with the GABA_A_ Receptor: Evidence for Overlapping Intersubunit Binding Modes

**DOI:** 10.1371/journal.pone.0142514

**Published:** 2015-11-03

**Authors:** Thomas Westergard, Reza Salari, Joseph V. Martin, Grace Brannigan

## Notice of Republication

This article was republished on October 21, 2015, to correct the name of the thyroid hormone in the title; “L-3,5,4'-Triiodothyronine” was corrected to read “L-3,5,3'-Triiodothyronine.” Please download this article again to view the correct version. The originally published, uncorrected article and the republished, corrected article are provided here for reference.

## Supporting Information

S1 File Originally published, uncorrected article. (PDF)

S2 File Republished, corrected article. (PDF)
