# Supplementary figures and images for: Interactions of L-3,5,3'-Triiodothyronine, Allopregnanolone, and Ivermectin with the GABAA Receptor: Evidence for Overlapping Intersubunit Binding Modes
Source: PLoS One. 2015 Sep 30;10(9):e0139072. doi: 10.1371/journal.pone.0139072 (PMC4589331; doi:10.1371/journal.pone.0139072)

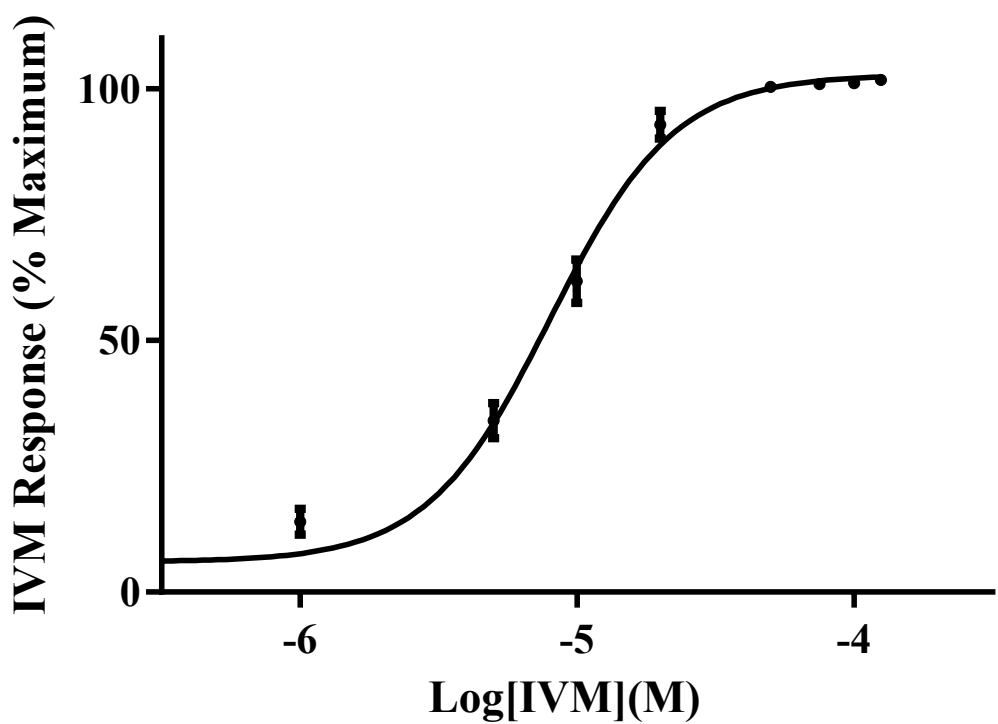

Supplement: S1 Fig — Data is fit to a Hill equation with EC50 of 7.1 ± 0.8 μM and Hill coefficient of 1.9 ± 0.4. Results are represented as a percentage of the maximal ivermectin response at 125 μM (mean ± S.E.M., n = 3). (PDF) [file pone.0139072.s001.pdf]

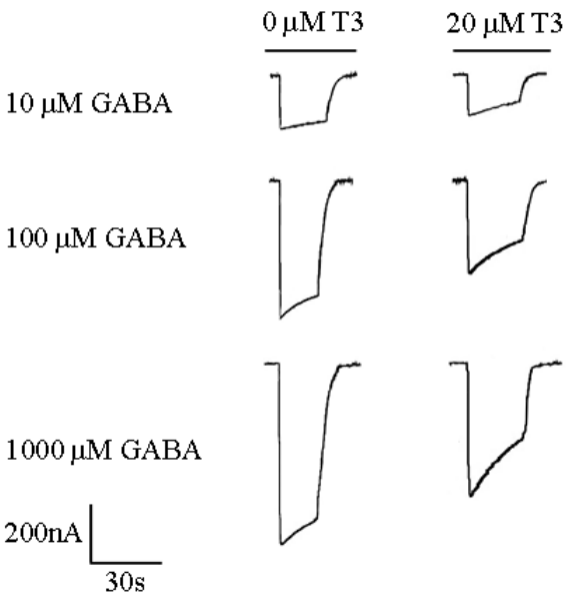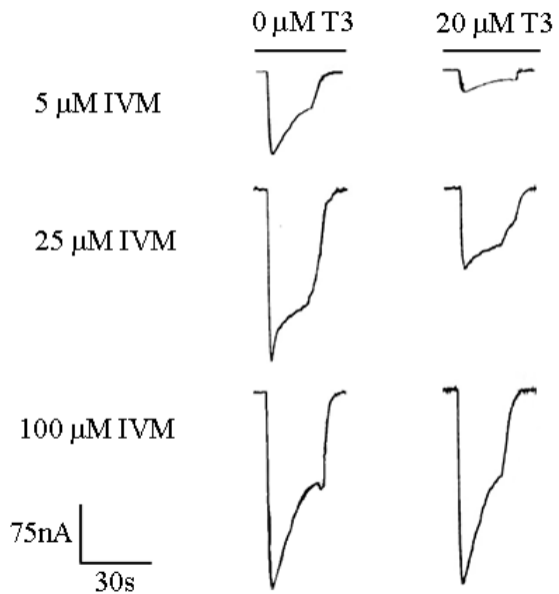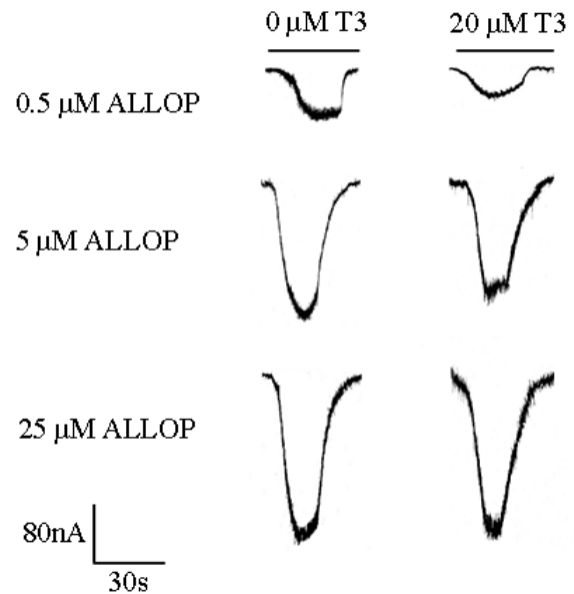

Supplement: S2 Fig — (PDF) [file pone.0139072.s002.pdf]

A

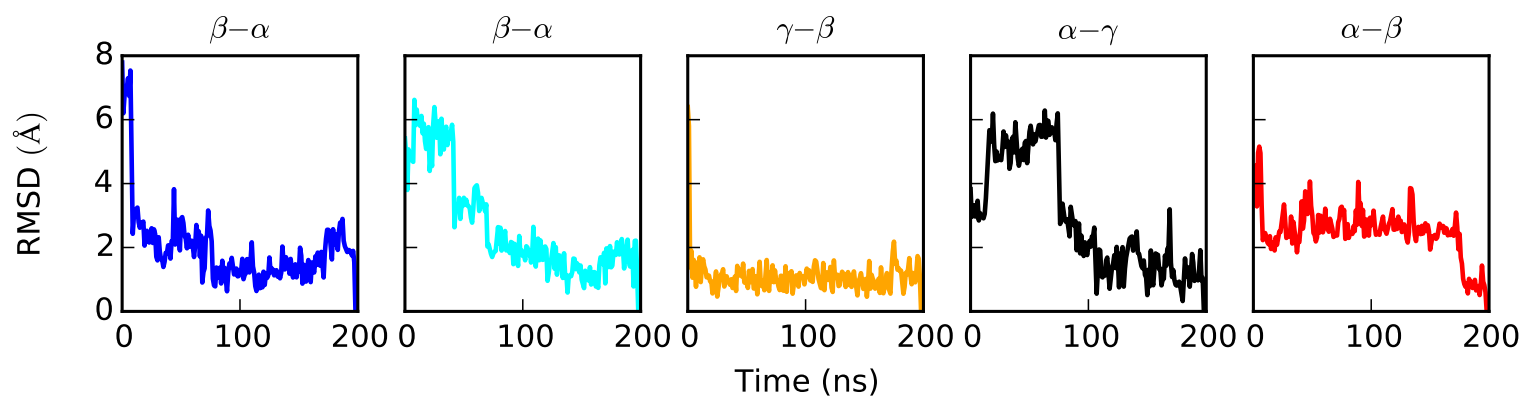

B

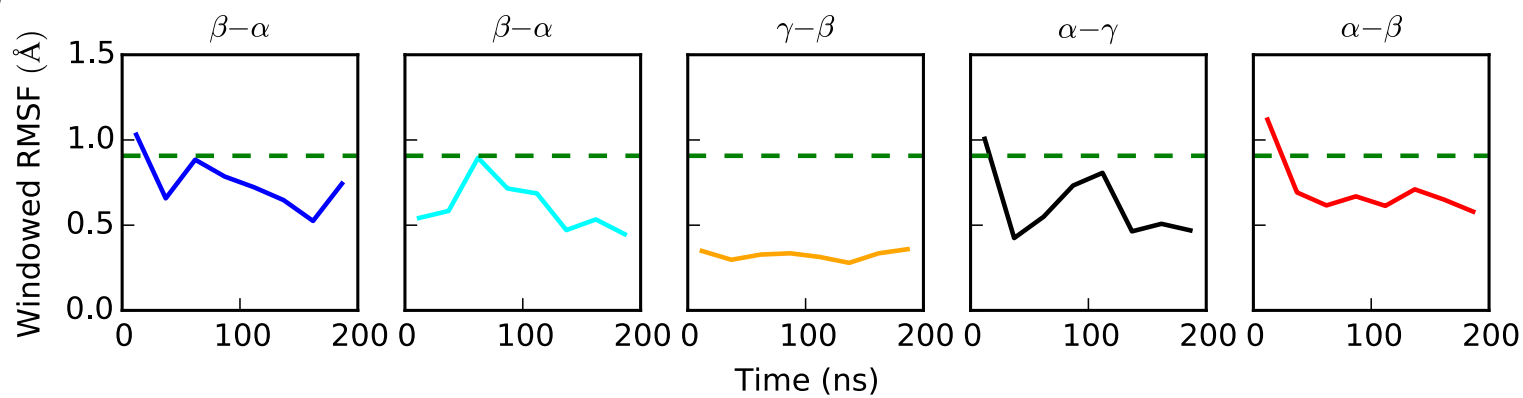

Supplement: S3 Fig — (A) Root-mean-squared-displacement (RMSD) trajectory for the carbon atoms of the T3 molecule in each interface is shown, relative to the last frame of the simulation. With the exception of the α-β interface, the RMSD decreased dramatically in the first 100 ns to at most 2Å and (with a few very short deviations) remained under 2Å for the remainder of the simulation. For the α-β interface, the T3 molecule unbound and spontaneously rebound. In both A) and B), the interfaces are ordered, left to right, in order of decreasing energetic favorability. (B) Root-mean-squared-fluctuation (RMSF) of all non-hydrogen atoms of T3 molecules bound to five subunit interfaces (solid lines) averaged over 8 consecutive 25 ns windows, as well as the equivalent RMSF for free T3 in water (green dashes). (PDF) [file pone.0139072.s003.pdf]

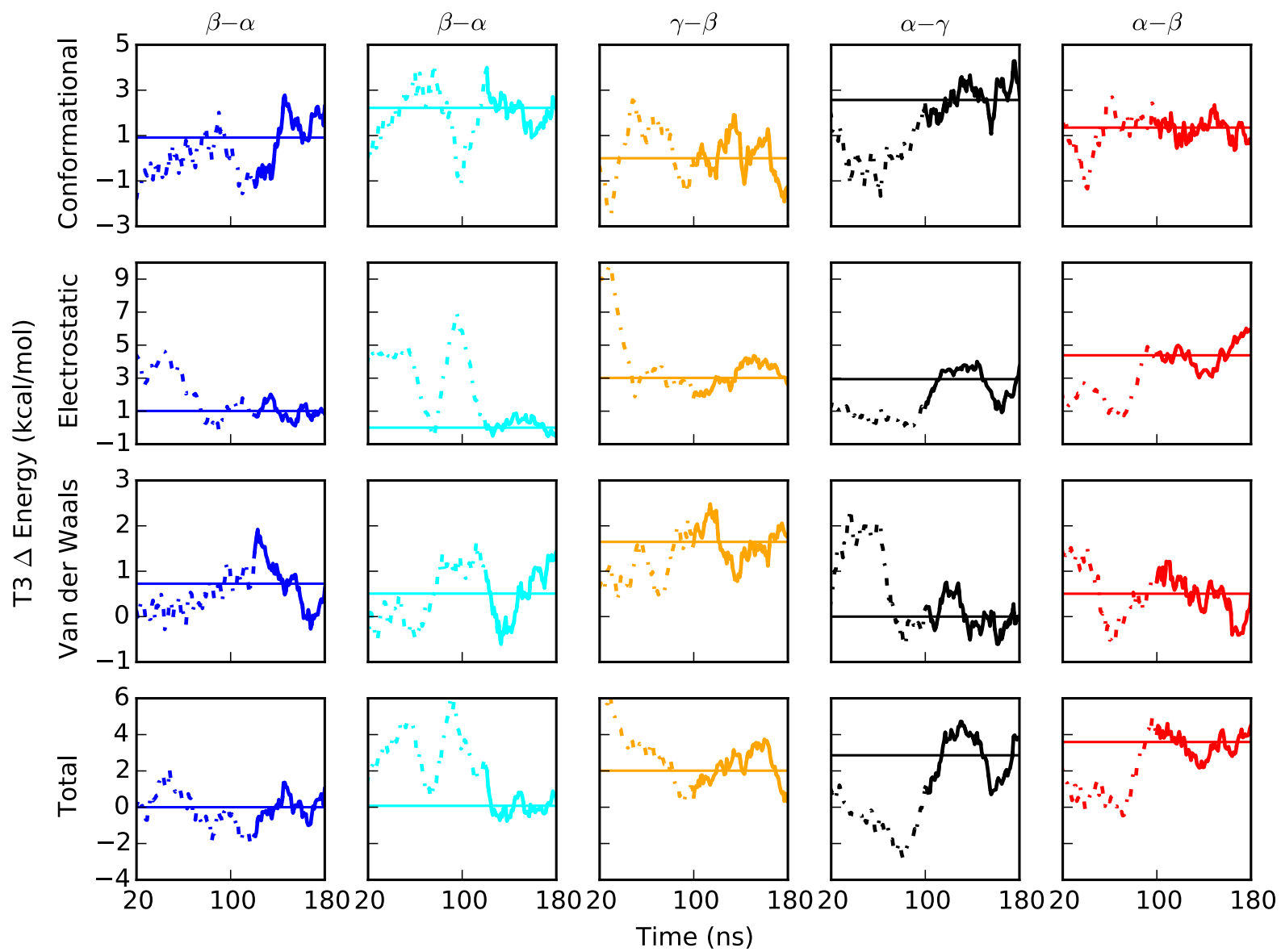

Supplement: S4 Fig — Energy trajectories were smoothed with a 20 ns window, with the initial and final 20 ns removed due to distortion from the windowing process. Solid region of the trajectory curve indicates the equilibrated regions used in calculating the averages represented by the horizontal solid line and listed in S2 Table. The final row showing the relative total energies is identical to Fig 6D. (PDF) [file pone.0139072.s004.pdf]
