# Supplementary material for: Interactions of L-3,5,3'-Triiodothyronine, Allopregnanolone, and Ivermectin with the GABAA Receptor: Evidence for Overlapping Intersubunit Binding Modes
Source: PLoS One. 2015 Sep 30;10(9):e0139072. doi: 10.1371/journal.pone.0139072 (PMC4589331; doi:10.1371/journal.pone.0139072)
Supplement: S1 Table — Lower values indicate a more favorable average score, and all values are shifted relative to the most favorable interface (underlined). Interfaces are listed from left to right in the order of favorability according to energy calculations from MD simulations (see Table 2). For T3, the affinity trend predicted by docking is reversed from the MD predicted trend, potentially due to underestimating favorability of ligand-backbone interactions in the docking calculations. No poses were identified for T3 in the γ-β and α-β interfaces. Autodock Vina docking scores are assigned units of kcal/mol. (PDF) [file pone.0139072.s009.pdf]

Ligand	β-α	β-α	γ-β	α-γ	α-β
T3	0.4	0.1	-	0	-
IVM	1.0	1.1	0	0.7	0.4
ALLOP	1.1	0.2	0.2	0	0.4
