# Supplementary material for: Interactions of L-3,5,3'-Triiodothyronine, Allopregnanolone, and Ivermectin with the GABAA Receptor: Evidence for Overlapping Intersubunit Binding Modes
Source: PLoS One. 2015 Sep 30;10(9):e0139072. doi: 10.1371/journal.pone.0139072 (PMC4589331; doi:10.1371/journal.pone.0139072)
Supplement: S2 Table — Lower values indicate a more favorable energy. Interfaces are listed from left to right in the order of favorability according to the final row. All values are in kcal/mol. (PDF) [file pone.0139072.s010.pdf]

Component	β-α	β-α	γ-β	α-γ	α-β
Bond	0.1	0.4	<u>0.0</u>	0.6	0.5
Angle	0.3	0.4	0.4	1.2	<u>0.0</u>
Dihedral	1.0	1.9	<u>0.0</u>	1.2	1.1
Improper	<u>0.0</u>	<u>0.0</u>	0.1	<u>0.0</u>	0.1
Total Conformational[1]	0.9	2.2	<u>0.0</u>	2.6	1.4
Electrostatic	1.0	<u>0.0</u>	3.0	2.9	4.4
van Der Waals	0.7	0.5	1.6	<u>0.0</u>	0.5
Total Non-bonded[1]	1.2	<u>0.0</u>	4.2	2.4	4.4
Total[1]	**<u>0.0</u>**	**0.1**	**2.0**	**2.9**	**3.6**

[1] The values in each row are offset so that the lowest energy interface (underlined) in the row has a value of 0, for consistency with Figures 6C and S4; consequently, the values in a given "Total" row may differ from the sum of the previous rows by a value that is constant across the interfaces.
