# Supplementary material for: Interactions of L-3,5,3'-Triiodothyronine, Allopregnanolone, and Ivermectin with the GABAA Receptor: Evidence for Overlapping Intersubunit Binding Modes
Source: PLoS One. 2015 Sep 30;10(9):e0139072. doi: 10.1371/journal.pone.0139072 (PMC4589331; doi:10.1371/journal.pone.0139072)
Supplement: S3 Table — All listed hydrogen bonds with non-polar residues represent interactions of the ligand with the carbonyl oxygen of the protein backbone at the location of a helix deformation in M1. No hydrogen bonds were identified in the α-β interface. Interfaces are listed from left to right in the order of favorability according to energy calculations from MD simulations (see S2 Table). (PDF) [file pone.0139072.s011.pdf]

	β-α	β-α	γ-β	α-γ	α-β
Avg Number / Frame[1]	0.6	1.1	0.8	0.9	0
Most Frequent[2]	αM1 I228 (40%)/ βM2 S265 (40%)	βM3 D282 (60%)	γM2 R284 (90%)	αM3 Q287 (70%)	-
Second Most Frequent[2]	βM2 T262 (10%)	αM1 Q229 (20%)	γM2 S280 (5%)	γ M1 I238 (30%)	-

1 Number of hydrogen bonds per frame formed between T3 and protein residues lining the cavity, averaged over the second 100 ns of the simulation; values greater than one indicate multiple hydrogen bonds formed in most frames.

2 Number in parentheses indicates the percentage of the average number/frame that was contributed by this interaction.
