# Supplementary material for: Correction: Interactions of L-3,5,3'-Triiodothyronine, Allopregnanolone, and Ivermectin with the GABAA Receptor: Evidence for Overlapping Intersubunit Binding Modes
Source: PLoS One. 2015 Nov 3;10(11):e0142514. doi: 10.1371/journal.pone.0142514 (PMC4631515; doi:10.1371/journal.pone.0142514)
Supplement: S1 File — (PDF) [file pone.0142514.s001.pdf]

RESEARCH ARTICLE

# Interactions of L-3,5,4'-Triiodothyronine, Allopregnanolone, and Ivermectin with the $GABA_A$ Receptor: Evidence for Overlapping Intersubunit Binding Modes

**Thomas Westergard[1,2], Reza Salari[2,3], Joseph V. Martin[2,4], Grace Brannigan[2,3]***

**1** Department of Neuroscience, Thomas Jefferson University, Philadelphia, Pennsylvania, United States of America, **2** Center for Computational and Integrative Biology, Rutgers University-Camden, Camden, New Jersey, United States of America, **3** Department of Physics, Rutgers University-Camden, Camden, New Jersey, United States of America, **4** Department of Biology, Rutgers University-Camden, Camden, New Jersey, United States of America

* grace.brannigan@rutgers.edu

CrossMark
click for updates

OPEN ACCESS

**Citation:** Westergard T, Salari R, Martin JV, Brannigan G (2015) Interactions of L-3,5,4'-Triiodothyronine, Allopregnanolone, and Ivermectin with the $GABA_A$ Receptor: Evidence for Overlapping Intersubunit Binding Modes. PLoS ONE 10(9): e0139072. doi:10.1371/journal.pone.0139072

**Editor:** James Porter, University of North Dakota, UNITED STATES

**Received:** June 9, 2015

**Accepted:** September 9, 2015

**Published:** September 30, 2015

**Copyright:** © 2015 Westergard et al. This is an open access article distributed under the terms of the Creative Commons Attribution License, which permits unrestricted use, distribution, and reproduction in any medium, provided the original author and source are credited.

**Data Availability Statement:** All relevant data are within the paper and its Supporting Information files.

**Funding:** This work was supported by National Science Foundation [MCB1330728], 'http://www.nsf.gov/div/index.jsp?div=MCB, JM, GB; National Science Foundation [DBI1126052], http://www.nsf.gov/div/index.jsp?div=DBI, TW, RS, GB; and National Science Foundation XSEDE program [MCB110149], https://www.xsede.org/, TW, GB.

**Competing Interests:** The authors have declared that no competing interests exist.

## Abstract

Structural mechanisms of modulation of γ-aminobutyric acid (GABA) type A receptors by neurosteroids and hormones remain unclear. The thyroid hormone L-3,5,3'-triiodothyronine (T3) inhibits $GABA_A$ receptors at micromolar concentrations and has common features with neurosteroids such as allopregnanolone (ALLOP). Here we use functional experiments on $\alpha_2\beta_1\gamma_2$ $GABA_A$ receptors expressed in *Xenopus* oocytes to detect competitive interactions between T3 and an agonist (ivermectin, IVM) with a crystallographically determined binding site at subunit interfaces in the transmembrane domain of a homologous receptor (glutamate-gated chloride channel, GluCl). T3 and ALLOP also show competitive effects, supporting the presence of both a T3 and ALLOP binding site at one or more subunit interfaces. Molecular dynamics (MD) simulations over 200 ns are used to investigate the dynamics and energetics of T3 in the identified intersubunit sites. In these simulations, T3 molecules occupying all intersubunit sites (with the exception of the α-β interface) display numerous energetically favorable conformations with multiple hydrogen bonding partners, including previously implicated polar/acidic sidechains and a structurally conserved deformation in the M1 backbone.

## Introduction

The ionotropic γ-aminobutyric acid (GABA) type A receptor ($GABA_A$) is a primary transducer of inhibitory signaling in the central nervous system. $GABA_A$ receptors are anionic members of the pentameric ligand-gated ion channel (pLGIC) or "Cys-loop receptor" superfamily. In addition to widely-used drugs like anesthetics [1–4] and sedatives [5,6], locally synthesized steroids called neurosteroids modulate $GABA_A$ receptors and act as endogenous sedatives,

anesthetics, analgesics, anti-convulsants, and anxiolytics. The neurosteroid 3α-hydroxy-5α-pregnan-20-one (allopregnanolone or ALLOP) potentiates response to GABA and can activate the receptor in the absence of GABA [7–10].

We have previously proposed [11] that the thyroid hormone, triiodothyronine (T3) modulates the $GABA_A$ receptor via a mechanism similar to that of neurosteroids. Such modulation has potential physiological significance as a non-genomic mechanism through which T3 exerts its significant effects on sleep and mood in adulthood [12–15]. T3 inhibits the activity of GABA-gated chloride currents on recombinant $GABA_A$ receptors expressed in human embryonic kidney-293 cells and *Xenopus* oocytes at lower concentrations [16], but activates at concentrations beyond 30 μM [16,17].

Similarities in the molecular dimensions and functional groups of T3 and neurosteroids are consistent with overlapping mechanisms [11] (Fig 1A), and the conformational flexibility of T3 is reduced by bulky iodine atoms (S1 Movie). Conformational flexibility of ALLOP is shown for comparison (S2 Movie 2). Structure-activity studies have demonstrated that, as in T3, true steroids must contain a hydroxyl at C3 and a hydrogen-bond accepting group at C20 in order to activate $GABA_A$ receptors [9,18,19]. Tetracyclic structure is not a strict requirement for binding [20] despite its presence in most identified endogenous lipophilic modulators of $GABA_A$ receptors. T3 is an endogenous novel dicyclic modulator [11] that may bind to the same sites as positively modulating neurosteroids but that causes negative modulation.

An extensively investigated group of sites for lipophilic modulators of eukaryotic pLGICs lie in pockets at the subunit interfaces, in the transmembrane domain. Significant evidence from mutagenesis and photoaffinity labeling approaches as well as molecular dynamics (MD) simulations suggests that anesthetics and alcohols bind to this region in the nicotinic acetylcholine receptor (nAChR), glycine receptor, and $GABA_A$ receptor [1,3,21–25]. Structural interpretation of results from site-directed mutagenesis and photoaffinity labeling has proven highly sensitive to details of the homology model used for the $GABA_A$ receptor. Earlier models were based on the cryo-electron microscopy structure [26] of the nAChR, which suffered from the ambiguous gap in the alignment between cationic and anionic pLGICs transmembrane domains; different alignments significantly alter the proposed orientation of many trans-membrane residues [27–29]

Site-directed mutagenesis [30,31] was used to identify two residues critical for activation of $GABA_A$ receptors ($\alpha_1$:M1:T237 and $\beta_2$:M1:Y284) by ALLOP; due to modeling ambiguities mentioned previously, it was not clear whether these residues formed a common binding pocket at the β-α (counter-clockwise) interface, as proposed in [30] or whether they faced into distinct intrasubunit cavities, as suggested in [29]. The alignment used for the model proposed in [29] was supported in part by evidence from cysteine mutagenesis and sulfhydryl cross-linking [27,28] and is also consistent with that used in newer models based on an unambiguous alignment with GluCl as well as the recent crystal structure [32] of a human $GABA_A$ β3 homopentamer. As a result, newer models are also consistent with the placement of these residues facing into distinct intrasubunit cavities, as originally proposed in [29]. Furthermore, ALLOP has been shown to enhance photoaffinity labeling of residues in the β-α subunit interface [29] by an etomidate analog, suggesting minimal competition between ALLOP and etomidate for that site, as well as the presence of at least one allosteric site for ALLOP distinct from the β-α subunit interfaces. The latter, however, has not been further confirmed, and other subunit interfaces have not been extensively investigated as potential allosteric binding sites for ALLOP.

Following site-directed mutagenesis studies [33,34], the recent crystal structure of GluCl [35] includes an intersubunit transmembrane site for an agonist (the antiparasitic IVM) that also activates the $GABA_A$ receptor [36,37]. While it is not confirmed that IVM occupies the

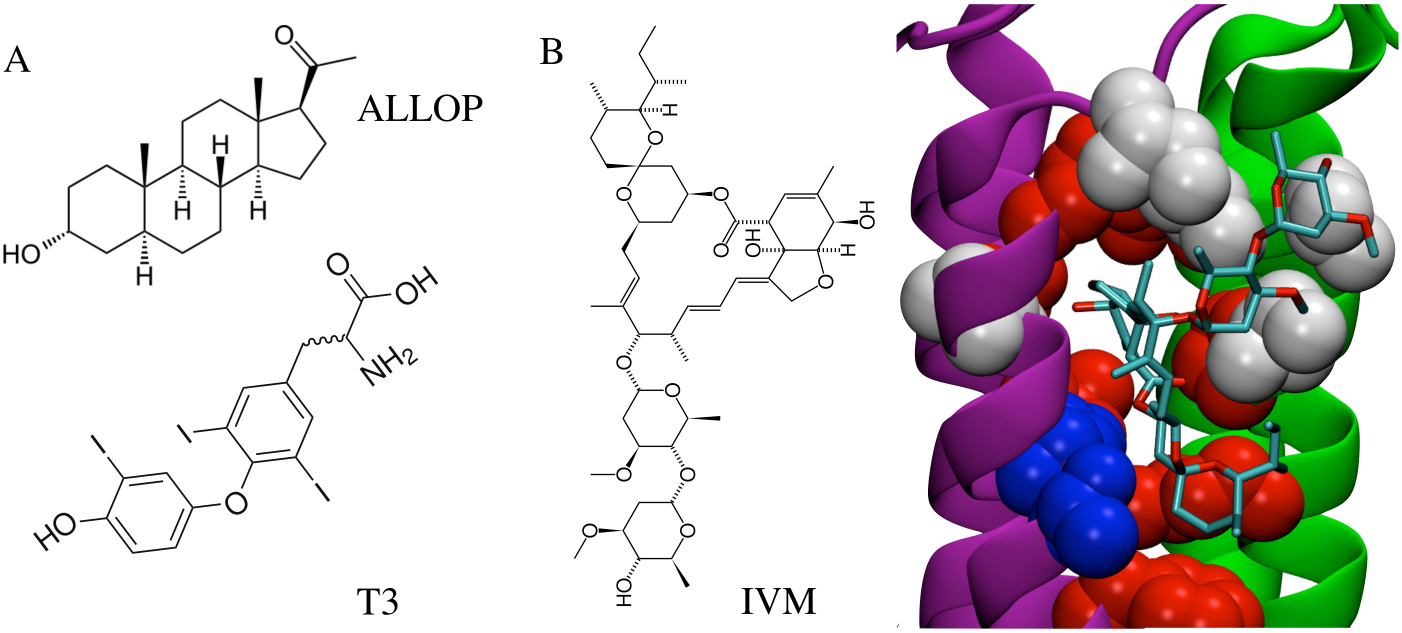


**Fig 1. Chemical analogy among modulators and receptors.** (A) Comparisons of structures of the neurosteroid ALLOP and thyroid hormone T3. Neuroactive steroids and T3 share common features, including molecular dimensions, placement of hydrogen-bond accepting groups, and multiple rings. (B) The GluCl IVM binding site. Residues identical to those at the $GABA_A$ β-α interface are red, similar residues are white, and residues with no similarity are blue.

doi:10.1371/journal.pone.0139072.g001

same binding mode in the $GABA_A$ receptor as it does in GluCl [33], the cavity is one of the regions with the closest homology (Fig 1B). Here we use competition of T3 and ALLOP with IVM as a test for likelihood of intersubunit binding. We focus on the intersubunit site due to its occupancy by IVM in the GluCl crystal structure and its previously identified significance for numerous modulators. We present here two-electrode voltage-clamp recordings and MD simulations used to explore this potential binding site for T3. We find that T3 acts competitively with IVM and with ALLOP, suggesting that T3 and ALLOP both bind to an intersubunit site. We perform docking calculations that identify intersubunit sites as potential sites for IVM, ALLOP, and T3. Over the course of a 200 ns MD simulation of T3 bound to a $GABA_A$ receptor model in a hydrated lipid bilayer, we find that T3 is stable in four intersubunit sites over these timescales, with spontaneous rebinding observed for the fifth interface. We further show that multiple T3 conformations, with distinct hydrogen binding partners including the M1 helix backbone, can have equivalently favorable energies. As described in *Discussion*, these observations suggest possible origins of inconclusive results for mutagenesis studies of various pLGIC allosteric modulators.

## Results

### Electrophysiological Studies

**Inhibition of GABA response by T3.** The inhibiting effect of T3 (0.1 μM- 100 μM) on $GABA_A$ receptor stimulation by 10 μM GABA is shown in Fig 2A, with a representative trace indicating a significant reduction in the response of GABA in the presence of 10 μM T3 (Fig 2A, inset). Further representative traces are in supplementary information (S2 Fig). Apparent-maximal concentrations of T3 (between 50–100 μM) reduced response to GABA to 60 ± 3% of control, with an $IC_{50}$ of 8 ± 2 μM (Fig 2A). These results are similar to previously reported

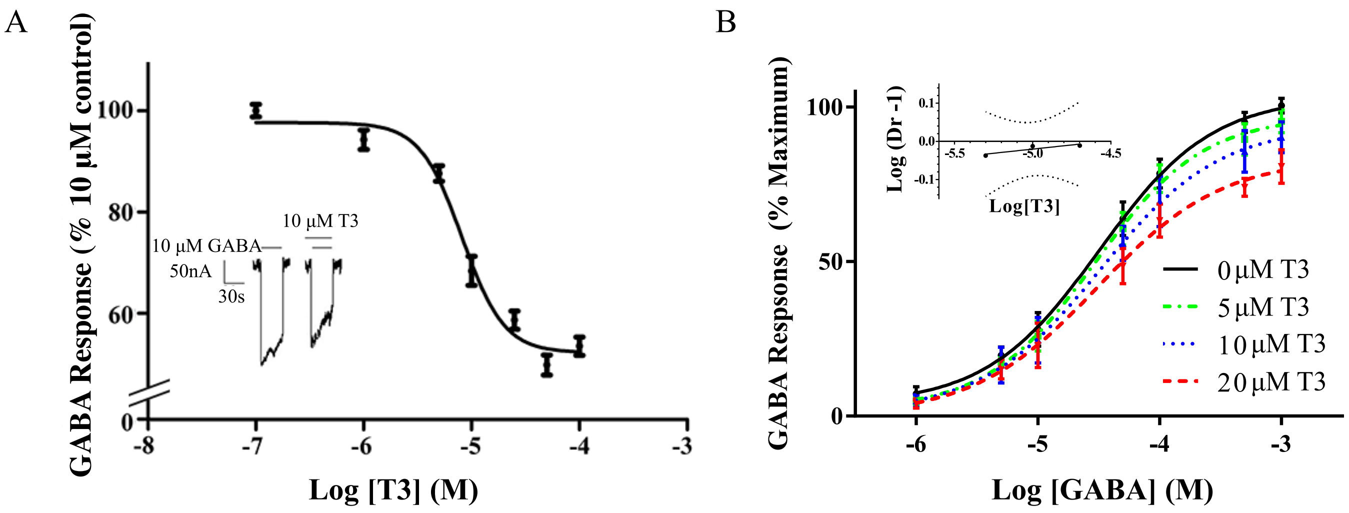


**Fig 2. Inhibition of GABA response by T3.** (A) Dose-response curve for the effects of T3 on the GABA-stimulated current as a percent of the maximal GABA response in the absence of T3. The values are expressed as a mean of three separate determinations, with error bars representing the standard error of the mean (S.E.M.). (A, Inset) Representative tracings for the effect of 10 µM GABA with or without added 10 µM T3. The solid lines above the tracing indicate the time of superfusion of the oocyte with the indicated solutions. (B) Evaluation of T3 inhibition of GABA response. Dose-response curves for the effects of GABA were constructed separately in the presence of 0, 5, 10, or 20 µM T3. The data are represented as means ± S.E.M. for triplicate determinations. For each data point, *n* = 3–5. (B, Inset) Schild plot of the data from (B). "Dr" stands for dose-ratio. The slope of the line was 0.04 ± 0.02, which was significantly different from unity according to 95% confidence levels (shown in dotted lines).

doi:10.1371/journal.pone.0139072.g002

findings using a $\alpha_1\beta_2\gamma_2$ construct expressed in Xenopus oocytes [17], indicating low sensitivity of T3 response to β subunit sequence, as also observed in neurosteroid response [38].

Varying concentrations of T3 were applied to evaluate the likelihood of competition between 1–1,000 µM GABA and T3. Resulting dose response curves had reduced Hill coefficients with increasing T3, inconsistent with a competitive interaction. The data were analyzed by the method of Schild [39] (Fig 2B, inset), yielding a Schild plot with a slope of 0.04 +/- 0.02, also inconsistent with a competitive effect. These results are consistent with our hypothesis that the lipophilic T3 molecule binds to a transmembrane binding site distinct from the extracellular GABA binding site, although transmembrane binding of T3 cannot be determined based on this result alone.

**T3 inhibition of activation by IVM.** Dose-response curves indicated that sufficient concentrations of IVM could activate the $\alpha_1\beta_1\gamma_2$ GABA$_A$ receptor in the absence of GABA, consistent with previous studies on $\alpha_1\beta_2\gamma_2$ GABA$_A$ receptors [40]. IVM concentrations ranging from 0.1 µM– 125 µM (S1 Fig) stimulated *Xenopus* oocytes expressing $\boldsymbol{\alpha_1\beta_2\gamma_2}$ constructs while uninjected oocytes were unaffected by IVM (data not shown). An apparent maximal effect of IVM was observed between 20–50 µM with an $EC_{50}$ of 7.1 ± 0.8 µM, and a Hill coefficient of 1.9 ± 0.4.

Activation of the GABA$_A$ receptor by IVM was inhibited by the presence of T3 (Fig 3A) with a representative tracing shown for 10 µM T3 (Fig 3A inset). Further representative traces are in supplementary information (S2 Fig). For the maximal concentrations of T3 used in this study (about 50 µM), response to IVM was reduced to 51% ± 5% of control, with an $IC_{50}$ for T3 of 7 ± 3 µM (Fig 3A); non-vanishing IVM response at 20 µM T3 likely corresponds to the onset of the high concentration regime associated with positive modulation and is addressed in the discussion.

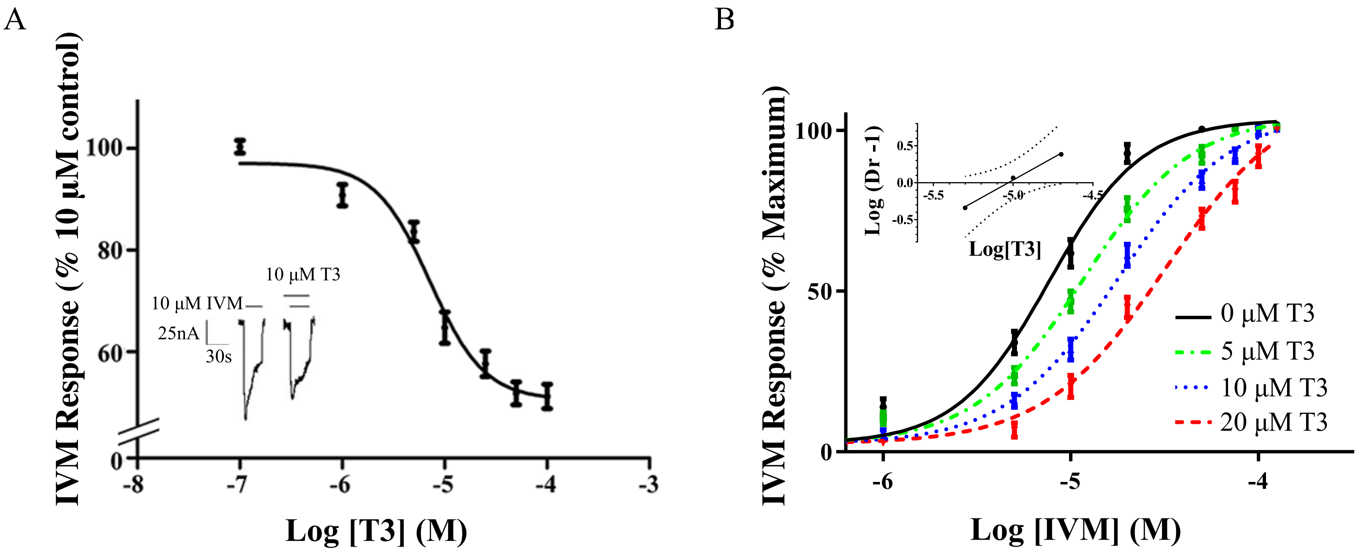


**Fig 3. Inhibition of IVM response by T3.** (A) Dose-response curve for the effects of T3 on the IVM-stimulated current as a percent of the maximal IVM response in the absence of T3. The values are expressed as a mean of three separate determinations ± S.E.M. (A, Inset) Representative tracings for the effect of 10 μM IVM with or without added 10 μM T3. The solid lines above the tracing indicate the time of superfusion of the oocyte with the indicated solutions. (B) Evaluation of T3 inhibition of the IVM response. Dose-response curves for the effects of IVM were constructed separately in the presence of 0, 5, 10, or 20 μM T3. The data are represented as means ± S.E.M. for triplicate determinations. For each data point, *n* = 3–5. (B, Inset) Schild plot of the data from (B). "Dr" stands for dose-ratio. The slope of the line was 1.2 ± 0.1, which was not significantly different from unity according to 95% confidence intervals, shown in dotted lines.

doi:10.1371/journal.pone.0139072.g003

**Competitive binding between T3 and IVM.** In order to evaluate the likelihood of competition between T3 and IVM for sites on the $GABA_A$ receptor transmembrane domain, the dose response to IVM was measured (Fig 3B) for various steady concentrations of T3 (0–20 μM). For this concentration range, increasing concentrations of T3 shifted curves to the right, maintaining the same slope but increasing $IC_{50}$ values. No changes were observed in the maximal response. A Schild analysis (Fig 3B inset) yielded a slope of 1.2 ± 0.1 with no significant difference from a slope of 1 according to 95% confidence intervals. Although the slope is not significantly different from one, it is still possible that the slight observed 0.2 deviation is not the result of statistical noise but instead reflects weak deviations of the mechanism from a simple competitive interaction. The shifted but parallel dose response curves with equivalent maximum responses shown in Fig 3B, however, further indicate that the dose ratio is insensitive to the concentration of agonist; such an observation would be surprising in a non-competitive inhibitory mechanism for which the agonist remains bound.

The Schild equation has been shown [41] to apply more generally to complicated models involving multiple agonist sites but which satisfy a basic set of assumptions. It is also thought to be approximately true for many cases in which those assumptions are weakly violated, such as the use of a partial agonist or inverse agonist rather than a true antagonist [41]. Many violations, including the presence of multiple binding sites for the antagonist, can be sufficiently strong to alter the intercept of the Schild plot without significantly changing the slope.

For the Schild plot shown in Fig 3B, the pA2 value calculated from the intercept was -5.0 ± 0.5 log concentration of T3 (about 10 μM), providing an estimated average affinity of T3 for inhibitory sites on the $GABA_A$ receptor that is consistent with the $IC_{50}$ for inhibition of $GABA_A$ receptor response by T3. We emphasize, however, that the relationship between the pA2 value and the average affinity will be less direct if inhibition occurs via multiple T3 sites with substantially different affinities and/or efficacies. The present estimates are consistent

with inhibition by T3 of both GABA- and IVM-induced currents via a common site(s), but are not sufficient to rule out multiple overlapping binding sites, with a subset inhibiting GABA induced currents and a non-overlapping or partially overlapping alternate subset inhibiting IVM-induced currents.

**T3 inhibition of activation by ALLOP.** Consistent with its well-known pharmacological effects on $GABA_A$ receptors, the neurosteroid ALLOP had a stimulatory effect on the $GABA_A$ receptor response in Xenopus oocytes (Fig 4), with an $EC_{50}$ of 0.9 ± 0.1 μM. For 1 μM ALLOP, this effect was inhibited 24% by the addition of 10 μM T3 in a sample tracing (Fig 4, lower inset). Further representative traces are in supplementary information (S2 Fig). Similar to the effect of T3 on stimulation by IVM, the addition of a constant concentration of T3 to a dose-response curve for ALLOP shifted the curve to the right. The Schild plot (Fig 4, upper inset) yielded a slope of 0.94±0.03, which was not significantly different from 1 according to 95% confidence intervals. The data therefore support the conclusion that T3 is a competitive inhibitor of the stimulatory effect of ALLOP at the $GABA_A$ receptor. A pA2 value of -4.7 ± 0.2 log concentration of T3 was calculated (about 20 μM), suggesting a value for the average affinity of T3 for the site(s) of competition. This value was consistent with the estimated affinity from competition between T3 and IVM, indicating that T3 might compete with both IVM and ALLOP via the same or similar sites; however it is not, in isolation, sufficient to conclude that all three sites overlap.

## Computational Results

**Docking calculations.** Docking calculations identified the subunit interface as a potential binding site for IVM, ALLOP, and T3 (Fig 5). Although these poses were likely facilitated by the cleft found in the GluCl template due to IVM binding in that structure, the asymmetry among docked poses suggests that the results are also sensitive to the receptor sequence. Asymmetry in docking scores (S1 Table) further suggests some degree of specificity, although the likely significance of these differences is reduced given dispersion in scores among identical sites (e.g. for ALLOP both the site with the least favorable and with the second most favorable average score were atβ- α interfaces). IVM and ALLOP were docked to all five subunits (each with a reduced average score for at least one of the β-α interfaces). T3 was only docked to the β-α and α- γ interfaces, with the latter having the most favorable score. This trend, however, was reversed in the MD simulations, as we discuss subsequently.

Unsurprisingly, orientation of IVM among the docked poses heavily overlapped with that in GluCl, with little dispersion among the poses within a given site. T3 poses were nearly all placed with the amino end of T3 closest to the pore-lining M2 helices and the hydroxyl end exposed to the lipid, while ALLOP was placed in multiple orientations with the hydroxyl either near to the M2 helices or facing the lipid. The increased dispersion in ALLOP poses relative to T3 poses likely reflects, in part, the bulky iodine atoms present in the latter molecule.

Although docking calculations primarily identified sites for T3 at the β-α and α- γ interfaces, false-negative results are common with docking to homology models with underdetermined placement of side-chains, so we cannot rule out potential sites at the other interfaces on this basis alone. Dispersion in resulting poses also yielded a certain amount of uncertainty in generating initial conditions for Molecular Dynamics Simulation. Finally, docking programs often underestimate the favorability of small molecule poses involving significant interactions with the protein backbone, which were observed for T3 in the MD simulations. Symmetric poses for initial conditions were obtained by alignment of similarly shaped T3 groups with IVM bound to sites on the $GABA_A$ receptor analogous to those in GluCl; this choice is further explained in *Methods*.

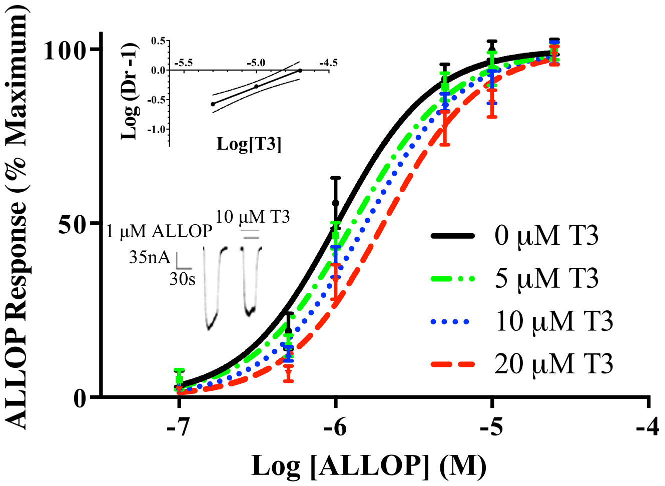


**Fig 4. Inhibition of ALLOP response by T3.** Dose-response curves for the effects of ALLOP were constructed separately in the presence of 0, 5, 10, or 20 μM T3. The data are represented as means ± S.E.M. for triplicate determinations. For each data point, *n* = 3–5. (Top Inset) Schild plot of the data from Fig 4. "Dr" stands for dose-ratio. The slope of the line was 1.2 ± 0.1, which was not significantly different from unity according to 95% confidence levels (shown in dotted line). (Bottom Inset) Representative tracings for the effect of 10 μM ALLOP with or without added 10 μM T3. The solid lines above the tracing indicate the time of superfusion of the oocyte with the indicated solutions.

doi:10.1371/journal.pone.0139072.g004

**Molecular dynamics of T3 in the intersubunit site.** Although more computationally expensive, MD simulations are able to provide insights beyond those of automated docking programs, by generating atomic resolution data on an ensemble of interactions between the parameterized ligand and a fully flexible binding site. The MD simulations used here take place over hundreds of nanoseconds, but are not likely to be sufficient for determining the effect of binding-site occupancy on global structure of the receptor, and are therefore not appropriate for inferring functional effects.

Initial (following minimization) and final poses of T3 (after 200 ns of Molecular Dynamics simulation) are shown in Fig 6A and 6B. Root-mean-squared-displacements (RMSD) between T3 in the initial and final frames ranged from 5.5–8.5 Å (S3 Fig, for comparison, an extended T3 molecule is about 15 Å in length). However, root-mean-squared-fluctuations (RMSF) for the heavy atoms of the T3 molecule over the last 100 ns of simulation ranged between 0.7 and 1.8 Å (S3 Fig), indicating that the T3 molecules become less fluid in the sites (and potentially more tightly bound) as the simulation proceeds.

The total energy of each bound T3 molecule was calculated over the course of the simulations (Fig 6D); this total included all intra-T3 contributions from conformational terms and non-bonded (van der Waals and electrostatic) interactions between atoms on the same T3 molecule, as well as any non-bonded interactions between T3 atoms and protein, water, lipid, or ion atoms. T3 molecules in the two β-α interfaces had equivalent average total energies, although as shown in S1–S3 Tables and S4 Fig, these reflected distinct conformations and hydrogen-bonding partners. In particular, T3 in the left-most β-α interface (Fig 6D) had conformational and non-bonded energies that were 1.3 kcal/mol more favorable and 1.2 kcal/mol less favorable, respectively, than that of the other β-α interface.

T3 molecules in the other interfaces had energies between 2 and 4 kcal/mol greater than in the β-α interface, in the following order from most to least favorable: γ-β (2kcal/mol), α-γ (2.9 kcal/mol), α-β (3.6 kcal/mol). Assuming that entropic costs of binding to each site are roughly equivalent, these results suggest that the γ-β site would be occupied at T3 concentrations of

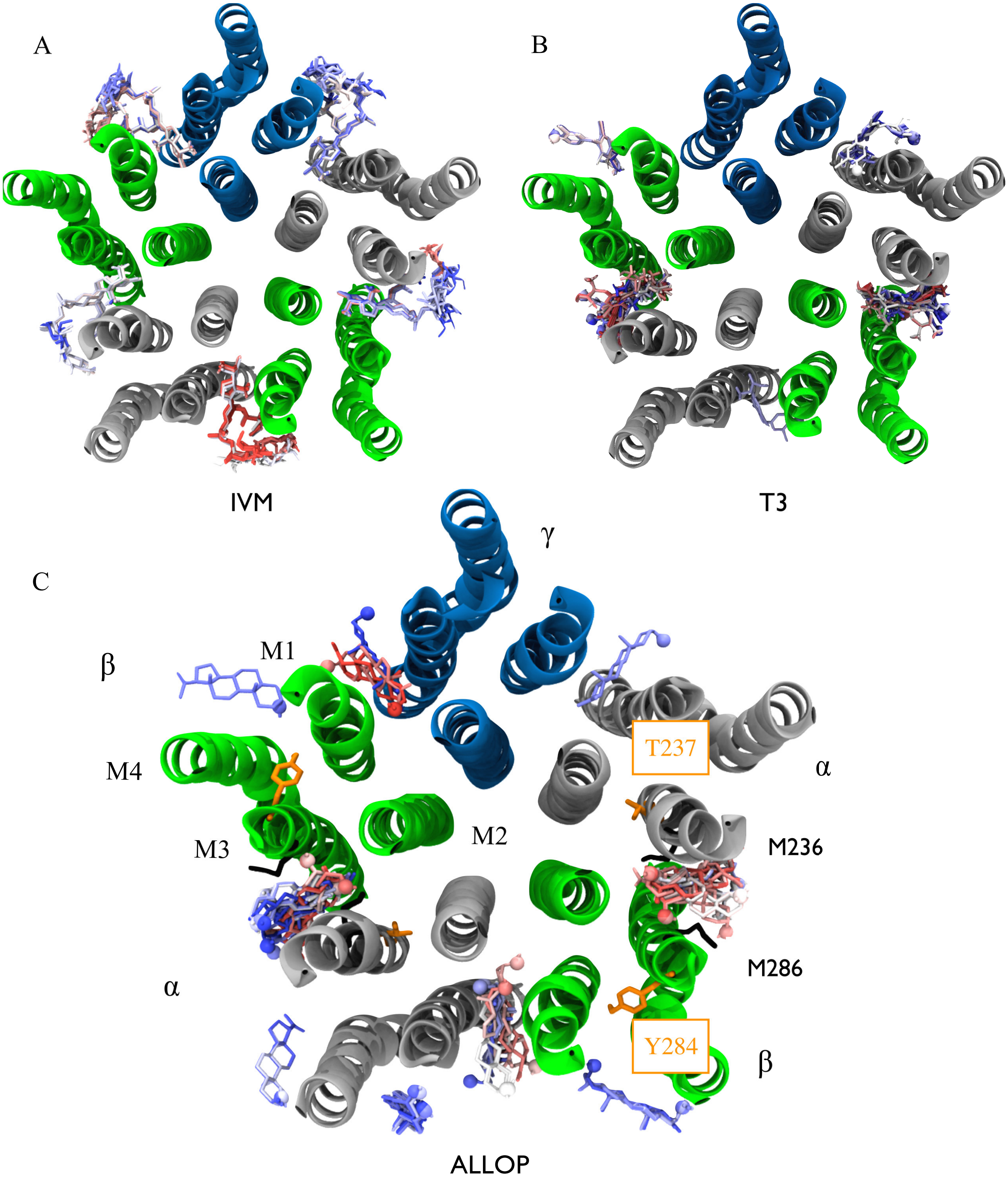


**Fig 5. Poses indicated by automated docking of IVM (A), T3 (B), and ALLOP (C) to the GABA$_A$ receptor model.** Five runs generating twenty poses each were conducted for all three ligands. Poses are colored according to docking score rank within a single run with strong scores in red, intermediate scores in white, and the weakest scores in blue, indicating that although poses for ALLOP and T3 are confined to subunit interfaces, multiple docking runs yield significant dispersion in orientation and ranking of individual interfaces (average scores in S1 Table). Poses located in the ion channel pore were excluded. The GABA$_A$ receptor transmembrane domain is shown and is colored by subunit: α-silver, β-green, γ-blue. In (B) and (C) the ligand hydroxyl is

shown as a space-filling sphere to indicate orientation. Residues implicated by mutagensis for activation by ALLOP are shown in orange (34) and those photolabeled by etomidate (35) are shown in black.

doi:10.1371/journal.pone.0139072.g005

about 30 ($10^{1.5}$) times that required for occupation of the β-α sites. For comparison, T3 inhibition of GABA stimulation by T3 is observable at 1 μM, but reverses at about 30–40 μM (Fig 2A and [17]).

With the exception of the least favorable (α-β) interface, T3 in all interfaces averaged between 0.6 and 1.1 hydrogen bonds per frame (S2 Table). The two β-α interfaces fell on either end of this extreme, with the interface in the first and second panel in Fig 6D averaging 0.6 and 1.1 hydrogen bonds per frame respectively. In the first case, 40% of the hydrogen bonds were formed between T3 and the backbone carbonyl oxygen of αM1 I228, at the location of a helix deformation that has been observed in all presently available high resolution crystal structures of pLGICs. An additional 40% were due to interactions with βM2 S265, a residue that

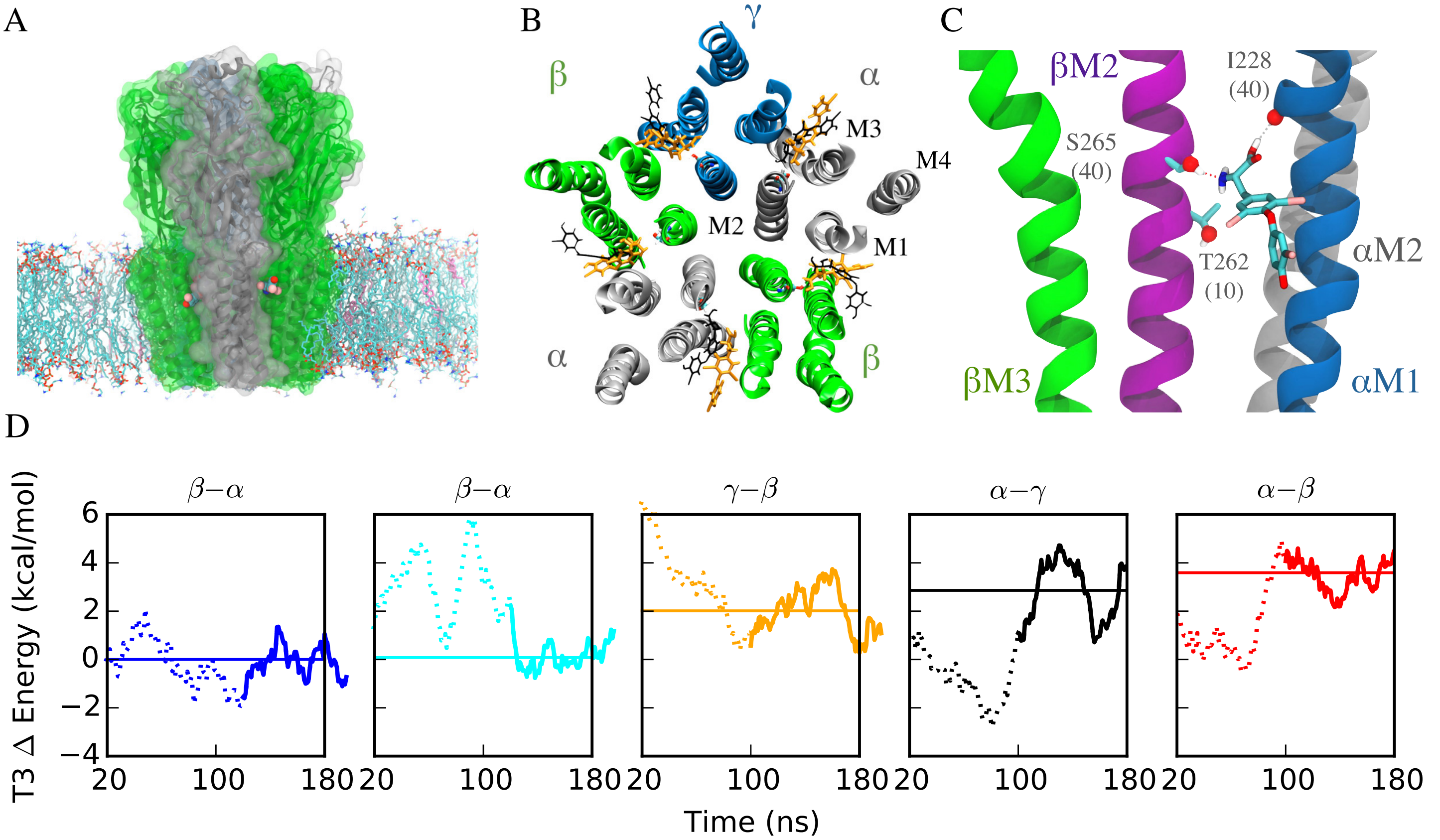


**Fig 6. Molecular Dynamics Simulations of $GABA_A$ receptors with T3 bound.** A) $GABA_A$ receptor-membrane complex, shown in cartoon with surface overlay, from a view parallel to the membrane. Lipids in the membrane are in licorice with POPC molecules colored by atom name and cholesterol molecules in pink. T3 molecules colored by atom name are shown at the interfaces between the α (silver) and β (green) subunits. B) Initial (gray) and final (orange) poses of T3 from 200 ns MD simulation. C) Representative frame showing interactions between T3 at one of the β-α interfaces, corresponding to the first column of S1–S3 Tables. Numbers shown in parentheses in residue labels represent the contribution of hydrogen bonds with the residue to the total number of hydrogen bonds observed in the second (equilibrated) half of the MD simulation (listed for all interfaces in S3 Table). T3 frequently formed simultaneous hydrogen bonds to αM1 I228 and βM2 S265, as shown here. D) Total energy of T3 molecules across trajectory, relative to that of the β-α interface shown in first column and in Panel C, and in left to right order of increasing average energy/decreasing favorability. Energy trajectories were smoothed with a 20 ns window, with the initial and final 20 ns removed due to distortion from the windowing process. Solid region of the trajectory curve indicates the equilibrated regions used in calculating the average listed in S2 Table, and represented here by the horizontal solid line. Decomposition of the total energies can be found in S2 Table and S4 Fig

doi:10.1371/journal.pone.0139072.g006

homologous to GluCl M2:S270, which forms a hydrogen bond with ivermectin in the GluCl crystal structure PDB:3RHW. These hydrogen bonds were formed simultaneously (as shown in Fig 6C) in about 75% of the frames in which they appeared. A third polar residue (βM2 T262) one turn toward the intracellular end of M2 contributed another 10% of the observed hydrogen bonds with T3.

In contrast, in the second β-α interface, T3 formed hydrogen bonds primarily as a donor with both acidic and polar sidechains: βM3 D282 and αM1 Q229, which contributed 60% and 20% of the observed average of 1.1 hydrogen bonds/frame respectively. While in both β-α interfaces, T3 served (primarily) as a donor to an M1 residue, in one interface T3 also served as an acceptor (primarily) to an M2 residue and in the other T3 served as a donor to an M3 residue; the average energy for T3 in each interface differed only by 0.1 kcal/mol. This result suggests that a number of binding modes in the same interface could be equally favorable for T3 (and possibly analogs like ALLOP), and consequently changes in affinity resulting from single residue substitutions may be particularly subtle.

## Discussion

The concentration of T3 in rat serum ranges from 1.6–1.9 nM [42,43]. However, thyroid hormone is taken up from blood and concentrated in brain tissue, particularly in the nerve terminals [44–46]. Synaptosomes, in a nerve-terminal fraction, are estimated to have concentrations of T3 ranging from 13.0–65 nM, depending on the methods used [47,48]. In disorders such as hypothyroidism, synaptosomes contain higher levels of T3 ranging to 126 nM, as part of a homeostatic mechanism to maintain brain hormone in the face of lowered circulating hormone [48]. The actual concentration of T3 at the site of the $GABA_A$ receptor in the synapse is difficult to evaluate, due in part to distributions within the membrane that are unlikely to be uniform. The sensitivity of the $GABA_A$ receptor to the lowest concentrations of thyroid hormone found in healthy humans was not specifically addressed in the current study, but other studies have shown effects of T3 in the hundred nanomolar range using other preparations [16]. T3 modulation of $GABA_A$ receptors therefore has a plausible significance in normal physiology, and apparent relevance to dysthyroid states. Concentrations of T3 tested in the current study are in the range of concentrations commonly used to demonstrate effects of pregnenolone sulfate [49]. While the effects of T3 show parallels to the effects of pregnenolone sulfate at 10 μM doses, there are interesting differences in the actions at higher 100 μM concentrations, where T3 has a stimulatory effect on the $GABA_A$ receptor [17]. Study of the effects of exposure to T3 is therefore likely to yield novel insight into the mechanisms of action of the $GABA_A$ receptor.

In the present work, we test the likelihood that T3 causes inhibition via a site that has been previously implicated in binding of positive modulators or agonists (IVM, general anesthetics, and activation by neurosteroids). We find that T3 reduces activation of $GABA_A$ receptors by IVM and ALLOP, with pharmacological analyses indicating competitive interactions. Furthermore, docking calculations return intersubunit transmembrane sites as potential T3 binding sites. In MD simulations, T3 molecules bound to these sites are stable over the 200 ns simulation time, and spontaneous rebinding is observed at the α-β interface. Our results are consequently consistent with an intersubunit binding site for IVM, T3, and ALLOP on the $GABA_A$ receptor.

The result that ALLOP may activate $GABA_A$ receptors via an intersubunit site is consistent with the results of Ref. [30,31]; however, the identified residues from that work are not expected to form this binding site, as suggested by Ref. [29]. Interpretation of the results of Ref. [30,31] and Ref. [29] is dependent upon the model used for the $GABA_A$ receptor [29], and

while the model used in [30] suggested that the two residues identified by mutagensis formed a common site, later models did not. These residues may have therefore been critical for transducing the effects of ALLOP and THDOC. The recent high-resolution structure [32] of a β3 homopentamer is consistent with more recent models indicating that implicated residues on the β subunit (Y284) face into the subunit center, rather than the subunit interface.

Persistence of IVM response at higher T3 concentrations is seemingly inconsistent with a simple binding mechanism. However, dose response to T3 consists of two regimes: about 1 to 30 μM, for which T3 negatively modulates IVM response, and above 30 μM, for which T3 acts as a positive modulator [17]. The present experimental study assumes that the low concentration regime can be treated approximately separately from the high concentration regime, due to expected negligible occupation of sites required for positive modulation at low concentrations, and results from this study are only presumed to be relevant to sites required for negative modulation. Interestingly, however, our energetic calculations from MD simulation suggest a difference of 2 kcal/mol in binding energy (a factor of 30 in dissociation constant) between the highest and second highest affinity interfacial sites; a mechanism in which the highest affinity (β-α) sites are inhibitory and the secondary site (γ-β) reverses the inhibitory effect would be quantitatively consistent with the onset of the high concentration potentiation regime (~30μM) given the onset of the low concentration inhibition regime (~1μM). Such a mechanism is also consistent with previous functional studies showing γ subunits to be unnecessary for inhibition of GABA by T3 [16].

Our focus on the intersubunit site in the present paper originates from the presence of IVM at the subunit interfaces in the GluCl homolog. We postulate here that IVM also activates $GABA_A$ receptors via some or all of the subunit interfaces; however, the role of each interface in the heterooligomer is unknown. Characterizing interface specificity for lipophilic ligands binding to pLGICs using experimental techniques has been particularly challenging, with pseudosymmetry posing well-defined challenges for crystallographic efforts and mutagenesis approaches yielding conflicting results. (For instance, efforts to interpret results of the βS265N mutation on general anesthetic sensitivity have spanned multiple decades; a recent discussion accompanies numerous new experiments to elucidate the effect's complex origins [50]). The latter may be due in part to the effects we observed in the present MD simulations, which suggest that multiple alternative protein-ligand interactions of equivalent strength may be capable of compensating for single residue substitutions. One such interaction observed here involves a hydrogen bond with the M1 helix backbone at a deformation conserved across available pLGIC structures, which puts a clear limitation on the power of a site-directed mutagenesis approach for altering affinity. If these predictions are accurate, techniques that introduce a steric block, either through substitutions with multiple bulky residues or competition with ligands of established interface specificity, as in [29], are likely to yield more useful results than those that aim to reduce affinity via weakened interactions with the cavity.

## Materials and Methods

### Experimental Studies

**Materials.** GABA, IVM, T3, and all other materials, unless stated otherwise, were obtained from Sigma (St. Louis, MO). T3 was initially dissolved in 0.1 M NaOH to make a stock concentrate of 1 mM T3, causing a slight 0.1 pH increase not expected to significantly affect $GABA_A$ receptor currents. During trials involving IVM, all solutions included 0.1% v/v dimethyl sulfoxide (DMSO), which was required due to the low solubility of the substances. This concentration of DMSO had no effect on the membrane properties of the oocytes.

The cDNAs encoding human $GABA_A$ receptor α1 and β1 subunits were acquired from American Type Culture Collection (ATCC, Manassas, Virginia), while those encoding γ2

subunits were acquired from GeneCopoeia (Rockville, Maryland). Restriction enzymes for the cDNAs came from New England Biolabs (Ipswich, MA).

Female *Xenopus laevis* were obtained from Xenopus Express Inc (Brooksville, FL). *X. laevis* were maintained according to Rutgers University Animal Care standards. The experimentation was approved by the Institutional Animal Care and Use Committee at Rutgers University.

**Synthesis of cRNAs.** *HindIII*-digested and *XholI*-digested DNA templates encoding human $\alpha_1$, $\beta_1$, or $\gamma_2$ $GABA_A$ receptor subunits (4 μg) were transcribed through the mMESSAGE mMACHINE T7 kit from Ambion (Austin, TX). Effects of neurosteroids are typically universal across known β subtypes [38]; use of the $\beta_1$ subtype rather than the previously used $\beta_2$ allowed us to test for similar universal effects for T3. The solution was then treated with RNase-free DNase I and precipitated with lithium chloride. The cRNAs were dissolved in double-distilled diethyl-pyrocarbonate (DEPC) treated water to a final concentration of ~ 1–2 μg/ μL. Subunit combinations were aliquoted for the specific receptor type with total concentration remaining ~ 1–2 μg/ μL.

**Oocyte preparation.** *X. laevis* were anesthetized by placing them in solution of 1 g tricaine methanesulfonate (MS222) per 500 mL in de-chlorinated water. A small incision in the abdomen was made 1 cm away from and parallel to the midline and lobes of ovary were removed. The lobes were gently agitated at room temperature with collagenase (2 mg/mL) in calcium free OR-2 (82.5 mM NaCl, 2.5 mM KCl, 1 mM $MgCl_2$, 5 mM HEPES, pH adjusted to 7.6 with NaOH) until the ovarian epithelium and the follicular cell layer were removed (~2 hrs). Oocytes were checked for proper size and shape, color, and for the presence of distinct animal and vegetal poles. Viable oocytes were placed in ND96 solution (96 mM NaCl, 2mM KCl, 1 mM $MgCl_2$, 1 mM $CaCl_2$, 5 mM HEPES, pH adjusted to 7.6 with NaOH) and the oocytes were injected with 46 nL cRNAs (1–2 μg/μL) expressing the $\alpha_1\beta_1\gamma_2$ construct using a digital micro-dispenser (Drummond 'Nanoject II'), with the ratio $\alpha_{1:}$ $\beta_{1:}$ $\gamma_2$ 1:1:2. Incorporation of the γ subunit was confirmed via potentiation of GABA response by diazepam. Injected oocytes were incubated at 18°C in a sterile solution containing Leibovitz L-15 Medium supplemented with 10 mM HEPES, 50 mg/L gentamicin and 50 mg/L tetracycline at pH 7.4.

**Two-electrode voltage-clamp recording.** After 24 h following injections, oocytes were placed in a recording chamber perfused continuously with ND96. All drugs were perfused into the chamber through a gravity flow system (~5 mL/min). Oocytes were impaled with two 3 M KCl-filled glass microelectrodes (~ 1–2 MΩ). Oocytes were then voltage-clamped at -60 mV with a Model OC-725C Oocyte Clamp (Warner Instruments, Hamden, CT). Currents were recorded using the iWorx LabScribe v1.959 software. The peak amplitudes of the responses were used for data analysis.

Trials began after five consecutive GABA, ALLOP or IVM responses had been observed to establish the peak amplitude of the response. In trials with T3, oocytes were preincubated with T3 (1–100 μM) for 15 s, followed directly with coapplication of T3 and GABA, ALLOP or IVM, for 30s. Oocytes were perfused with ND96 between trials until current returned to baseline (approximately 10–15 minutes). The responses are expressed as a percentage of the peak amplitude of response without T3.

During the competition studies, concentration-response curves of GABA, IVM, or ALLOP (0.1–125 μM) were determined against steady concentrations of T3 (0–20 μM). The pre-trial response to 125 μM GABA, IVM, or ALLOP without T3 was used as the maximum response to which values were compared.

## Computational Studies

**$GABA_A$ receptor model.** Docking and simulations used a previously built [51] model of the $\alpha_1\beta_1\gamma_2$ $GABA_A$ receptor, constructed using the structure for GluCl from *C. elegans* (PDB

code:3RHW) as a template, as well as the accompanying alignment [35]. The template is presumed to be in the open state due to the presence of an agonist (IVM) and the dimensions of the pore. The simulations conducted here do not include IVM or GABA; however, the IVM cavity found in the template persists to the initial structure for the model. The native structure of the cavity in the absence of IVM was unknown at the time of these simulations, but thought to be partially closed [52] and/or partially filled with lipid tails or cholesterol [51] that could exchange with other modulators; recent crystal structures [53] in the absence of IVM have illustrated both of these scenarios.

The model was built using MODELLER [54] in the order $\gamma_2$ $\beta_1$ $\alpha_1$ $\beta_1$ $\alpha_1$ (counter-clockwise), based on the results from Ref. [55]. Homology models of the $GABA_A$ receptor based on GluCl benefit from the unambiguous (no gaps) alignment of the two sequences in the transmembrane domain as well as the highly conserved fold in the transmembrane domain, as indicated by very close overlap of the protein backbones for prokaryotic GLIC and eukaryotic GluCl.

**Ligand coordinates.** Autodock Tools [56] was used for docking search space and ligand preparation. Automated docking of IVM, ALLOP, and T3 to the complete $GABA_A$ receptor model was conducted using AutoDock Vina [57]. Five runs generating twenty conformations (poses) of the ligand each were calculated with the exhaustiveness parameter that controls the extent of each search set to 20, well beyond the default value of 8; poses located in the ion channel pore (about 1–4 per run) were removed. No poses in the extracellular domain or in the subunit centers of the transmembrane domain were identified by the algorithm, for any of the three modulators. Use of flexible residues was limited to βM227 (which was required for symmetric docking of IVM) since subsequent MD simulations were expected to provide significantly more extensive and realistic flexibility than that accessible through automated docking.

For the MD simulations, initial coordinates were determined from an alignment of T3 with IVM so that T3 was placed symmetrically in all binding pockets. While likely not ideal as a method for estimating actual coordinates of the binding mode, this approach was considered preferable for providing initial coordinates to be relaxed during the simulation, since groups in identified poses for T3 did overlap similar groups in IVM in most poses, but with significant dispersion. As a result, any resulting asymmetry in relaxation of the binding interactions was not biased by potentially artificial asymmetries in initial coordinates.

**Molecular dynamics simulation.** The $GABA_A$/T3 complex was placed into a 12 x 12 $nm^2$ lipid bilayer with a 3 to 1 concentration of 1-palmitoyl-2-oleoyl-sn-glycerol-phosphatidyl-choline (POPC) and cholesterol using VMD [58]. The system was fully solvated to a box size of 12 x 12 x 18 $nm^3$ using the SOLVATE plugin and ionized with sodium and chloride ions for a 0.15 M neutralizing solution using the AUTOIONIZE plugin.

All simulations were run using the NAMD2.9 package [59]. The CHARMM22-CMAP force field was used for proteins [60] and CHARMM36 was used for the phospholipids, ions, and water [61]. The CHARMM generalized forcefield [62] was used for T3, with parameters generated via the ParamChem Server [63] with low penalties on all values. Periodic boundary conditions were applied, with particle-mesh Ewald long-range electrostatics and a cut-off of 1.2 nm Lennard-Jones potentials, with a smooth switching function starting at 1.0 nm. The simulations were run at a temperature of 300 K, pressure of 1 atm, and vanishing surface tension. Simulations began with 5 ns of simulation with harmonic restraints of 1 kcal/mol/Å on the Cα atoms of the protein. The restraints were then lifted and the simulation was run for another 195 ns. All MD runs were performed on the either the Cray XT5 supercomputer Kraken at the National Institute for Computational Sciences, University of Tennessee at Knoxville, TN or the Intel EM64T Xeon E5 cluster Gordon at the San Diego Supercomputer Center.

**Data analysis.** Graph Pad Prism, version 5.00 (La Jolla, CA), was used for all statistical analysis of two-electrode voltage-clamp data and for fitting the curves by nonlinear regression

for determinations of $EC_{50}/IC_{50}$, min and max response values, and Hill coefficients. To determine the type of competition, a Schild plot analysis was applied to the competitive binding data, utilizing a linear regression fit [39]. The agonist dose response $EC_{50}$ values at various T3 concentrations were compared to the $EC_{50}$ value with no T3 present, and then plotted against the log concentration of T3. The slope and x-intercept were used to analyze the type of competition and an estimate of the $K_d$ for T3.

Analysis of MD trajectories was performed using scripts written in VMD [58]. Hydrogen bonds were measured using a relatively loose definition, designed to detect weak hydrogen bonds; they were defined as those with an O/N-H separation less than 3.3 Å and an angle less than 25°. Graphing and smoothing of MD data was performed using scripts in Python 2.7.

## Supporting Information

**S1 Fig. Activation of $\alpha_1\beta_1\gamma_2$ $GABA_A$ receptor by ivermectin (IVM).** Data is fit to a Hill equation with $EC_{50}$ of 7.1 ± 0.8 μM and Hill coefficient of 1.9 ± 0.4. Results are represented as a percentage of the maximal ivermectin response at 125 μM (mean ± S.E.M., n = 3).
(PDF)

**S2 Fig. Representative traces for T3 modulation of $\alpha_1\beta_1\gamma_2$ $GABA_A$ receptor response to GABA (left), IVM (center), ALLOP (right).**
(PDF)

**S3 Fig. Dynamics of Bound and Free T3 molecules from Molecular Dynamics Simulation.** **(A)** Root-mean-squared-displacement (RMSD) trajectory for the carbon atoms of the T3 molecule in each interface is shown, relative to the *last* frame of the simulation. With the exception of the α-β interface, the RMSD decreased dramatically in the first 100 ns to at most 2Å and (with a few very short deviations) remained under 2Å for the remainder of the simulation. For the α-β interface, the T3 molecule unbound and spontaneously rebound. In both A) and B), the interfaces are ordered, left to right, in order of decreasing energetic favorability. **(B)** Root-mean-squared-fluctuation (RMSF) of all non-hydrogen atoms of T3 molecules bound to five subunit interfaces (solid lines) averaged over 8 consecutive 25 ns windows, as well as the equivalent RMSF for free T3 in water (green dashes).
(PDF)

**S4 Fig. Trajectory of T3 potential energy contributions for each interface, relative to interface with minimum average T3 energy for a given contribution.** Energy trajectories were smoothed with a 20 ns window, with the initial and final 20 ns removed due to distortion from the windowing process. Solid region of the trajectory curve indicates the equilibrated regions used in calculating the averages represented by the horizontal solid line and listed in S2 Table. The final row showing the relative total energies is identical to Fig 6D.
(PDF)

**S1 Movie. Molecular Dynamics simulation of one T3 molecule in water (10 ns).**
(MPG)

**S2 Movie. Molecular Dynamics simulation of one ALLOP molecule in water (10 ns).**
(MPG)

**S3 Movie. Molecular Dynamics simulation of five T3 molecules bound to intersubunit sites.** The transmembrane domain (viewed from the extracellular domain) is shown, with the protein colored by subunit: α (silver), β (green), γ (blue). T3 molecules are colored to

correspond with Fig 6C; the red T3 molecule at the γ-β interface shows a brief unbinding event before rebinding.
(MPG)

**S4 Movie. Molecular Dynamics simulation of five T3 molecules bound to intersubunit sites.** The transmembrane domain (viewed laterally from within the membrane) is shown, with the protein colored as in S3 Movie.
(MPG)

**S1 Table. Relative Autodock Vina docking score for ligand in each interface, averaged across 5 runs of 20 poses each.** Lower values indicate a more favorable average score, and all values are shifted relative to the most favorable interface (underlined). Interfaces are listed from left to right in the order of favorability according to energy calculations from MD simulations (see Table 2). For T3, the affinity trend predicted by docking is reversed from the MD predicted trend, potentially due to underestimating favorability of ligand-backbone interactions in the docking calculations. No poses were identified for T3 in the γ-β and α-β interfaces. Autodock Vina docking scores are assigned units of kcal/mol.
(PDF)

**S2 Table. Average Potential Energy of T3 over second half of MD simulation (100 ns), decomposed into contributions from separate terms, relative to the interface with the lowest average for a given component.** Lower values indicate a more favorable energy. Interfaces are listed from left to right in the order of favorability according to the final row. All values are in kcal/mol.
(PDF)

**S3 Table. Frequency of T3 hydrogen bond formation and common hydrogen-bonding partners during MD simulation.** All listed hydrogen bonds with non-polar residues represent interactions of the ligand with the carbonyl oxygen of the protein backbone at the location of a helix deformation in M1. No hydrogen bonds were identified in the α-β interface. Interfaces are listed from left to right in the order of favorability according to energy calculations from MD simulations (see S2 Table).
(PDF)

## Acknowledgments

The authors thank Dr. Jerome Henin of Institut de Biologie Physico-Chimique, Centre national de la recherche scientifique (CNRS) for helpful discussions. This research was funded by a grant from the National Science Foundation [MCB1330728], and was supported with computational resources from the National Science Foundation XSEDE program [MCB110149] as well as a local cluster funded by the National Science Foundation [DBI1126052].

## Author Contributions

Conceived and designed the experiments: TW JM GB. Performed the experiments: TW. Analyzed the data: TW RS GB. Wrote the paper: TW JM GB. Wrote python or VMD scripts used in analysis of computational data: RS GB.

## References

1. Hemmings HCJ, Akabas MH, Goldstein PA, Trudell JR, Orser BA, Harrison NL (2005) Emerging molecular mechanisms of general anesthetic action. Trends Pharmacol Sci 26: 503–510. PMID: 16126282

2. Miller KW (2002) The nature of sites of general anaesthetic action. Br J Anaesth 89: 17–31. PMID: 12173229
3. Krasowski MD, Harrison NL (1999) General anaesthetic actions on ligand-gated ion channels. Cell Mol Life Sci 55: 1278–1303. PMID: 10487207
4. Garcia PS, Kolesky SE, Jenkins A (2010) General anesthetic actions on GABAA receptors. Current neuropharmacology 8: 2. doi: 10.2174/157015910790909502 PMID: 20808541
5. Olsen RW, Sieghart W (2009) $GABA_A$ receptors: Subtypes provide diversity of function and pharmacology. Neuropharmacology 56: 141–148. doi: 10.1016/j.neuropharm.2008.07.045 PMID: 18760291
6. Rudolph U, Möhler H (2006) GABA-based therapeutic approaches: $GABA_A$ receptor subtype functions. Current opinion in pharmacology 6: 18–23. PMID: 16376150
7. Mitchell EA, Herd MB, Gunn BG, Lambert JJ, Belelli D (2008) Neurosteroid modulation of $GABA_A$ receptors: Molecular determinants and significance in health and disease. Neurochemistry international 52: 588–595. PMID: 18055067
8. Lambert JJ, Cooper MA, Simmons RDJ, Weir CJ, Belelli D (2009) Neurosteroids: Endogenous allosteric modulators of $GABA_A$ receptors. Psychoneuroendocrinology 34: S48–S58. doi: 10.1016/j.psyneuen.2009.08.009 PMID: 19758761
9. Akk G, Covey DF, Evers AS, Steinbach JH, Zorumski CF, Mennerick S (2007) Mechanisms of neurosteroid interactions with GABAA receptors. Pharmacology & Therapeutics 116: 35–57.
10. Belelli D, Lambert JJ (2005) Neurosteroids: endogenous regulators of the GABA(A) receptor. Nat Rev Neurosci 6: 565–575. PMID: 15959466
11. Martin JV, Padron JM, Newman MA, Chapell R, Leidenheimer NJ, Burke LA (2004) Inhibition of the activity of the native γ-aminobutyric acidA receptor by metabolites of thyroid hormones: correlations with molecular modeling studies. Brain Research 1004: 98–107. PMID: 15033424
12. Dratman MB, Gordon JT (1996) Thyroid hormones as neurotransmitters. Thyroid 6: 639–647. PMID: 9001201
13. Martin JV, Giannopoulos PF, Moffett SX, James TD (2013) Effects of acute microinjections of thyroid hormone to the preoptic region of euthyroid adult male rats on sleep and motor activity. Brain research 1516: 45–54. doi: 10.1016/j.brainres.2013.01.032 PMID: 23348377
14. Moffett SX, Giannopoulos PF, James TD, Martin JV (2013) Effects of acute microinjections of thyroid hormone to the preoptic region of hypothyroid adult male rats on sleep, motor activity and body temperature. Brain research 1516: 55–65. doi: 10.1016/j.brainres.2013.04.017 PMID: 23603414
15. Hu LY, Shen CC, Hu YW, Chen MH, Tsai CF (2013) Hyperthyroidism and Risk for Bipolar Disorders: A Nationwide Population-Based Study. PloS one 8: e7305.
16. Martin J, Williams D, Fitzgerald R, Im H, Vonvoigtlander P (1996) Thyroid hormonal modulation of the binding and activity of the GABAA receptor complex of brain. Neuroscience 73: 705–713. PMID: 8809792
17. Chapell R, Martin J, Machu TK, Leidenheimer NJ (1998) Direct channel-gating and modulatory effects of triiodothyronine on recombinant GABAA receptors. European journal of Pharmacology 349: 115–121. PMID: 9669504
18. Covey DF, Evers AS, Mennerick S, Zorumski CF, Purdy RH- (2001) Recent developments in structure–activity relationships for steroid modulators of GABAA receptors. Brain Research Reviews 37: 91–97. PMID: 11744077
19. Chisari M, Eisenman LN, Covey DF, Mennerick S, Zorumski CF (2010) The sticky issue of neurosteroids and GABAA receptors. Trends in Neurosciences 33: 299–306. doi: 10.1016/j.tins.2010.03.005 PMID: 20409596
20. Hu Y, Zorumski CF, Covey DF (1993) Neurosteroid analogs: structure-activity studies of benz[e]indene modulators of GABAA receptor function. 1. The effect of 6-methyl substitution on the electrophysiological activity of 7-substituted benz[e]indene-3-carbonitriles. Journal of Medicinal Chemistry 36: 3956–3967. PMID: 8254624
21. Brannigan G, LeBard DN, Hénin J, Eckenhoff RG, Klein ML (2010) Multiple binding sites for the general anesthetic isoflurane identified in the nicotinic acetylcholine receptor transmembrane domain. Proceedings of the National Academy of Sciences of the United States of America 107: 14122–14127. doi: 10.1073/pnas.1008534107 PMID: 20660787
22. Chiara DC, Dangott LJ, Eckenhoff RG, Cohen JB (2003) Identification of nicotinic acetylcholine receptor amino acids photolabeled by the volatile anesthetic halothane. Biochemistry 42: 13457–13467. PMID: 14621991
23. Nirthanan S, Garcia G, Chiara DC, Husain SS, Cohen JB (2008) Identification of binding sites in the nicotinic acetylcholine receptor for TDBzl-etomidate, a photoreactive positive allosteric effector. J Biol Chem 283: 22051–22062. doi: 10.1074/jbc.M801332200 PMID: 18524766

24. Belelli D, Pistis M, Peters JA, Lambert JJ (1999) The interaction of general anaesthetics and neurosteroids with GABA(A) and glycine receptors. Neurochem Int 34: 447–452. PMID: 10397373
25. Murail S, Wallner B, Trudell JR, Bertaccini E, Lindahl E (2011) Microsecond simulations indicate that ethanol binds between subunits and could stabilize an open-state model of a glycine receptor. Biophys J 100: 1642–1650. doi: 10.1016/j.bpj.2011.02.032 PMID: 21463577
26. Unwin N (2005) Refined Structure of the Nicotinic Acetylcholine Receptor at 4 Å Resolution. Journal of molecular biology 346: 967–989. PMID: 15701510
27. Jansen M, Bali M, Akabas MH (2008) Modular design of Cys-loop ligand-gated ion channels: functional 5-HT3 and GABA rho1 receptors lacking the large cytoplasmic M3M4 loop. J Gen Physiol 131: 137–146. doi: 10.1085/jgp.200709896 PMID: 18227272
28. Jansen M, Akabas MH (2006) State-dependent cross-linking of the M2 and M3 segments: functional basis for the alignment of GABAA and acetylcholine receptor M3 segments. The Journal of neuroscience 26: 4492–4499. PMID: 16641228
29. Li G-D, Chiara DC, Cohen JB, Olsen RW (2009) Neurosteroids allosterically modulate binding of the anesthetic etomidate to γ-aminobutyric acid type A receptors. Journal of Biological Chemistry 284: 11771–11775. doi: 10.1074/jbc.C900016200 PMID: 19282280
30. Hosie AM, Wilkins ME, da Silva HMA, Smart TG (2006) Endogenous neurosteroids regulate GABAA receptors through two discrete transmembrane sites. Nature 444: 486–489. PMID: 17108970
31. Hosie AM, Wilkins ME, Smart TG (2007) Neurosteroid binding sites on GABAA receptors. Pharmacology & Therapeutics 116: 7–19.
32. Miller PS, Aricescu AR (2014) Crystal structure of a human GABAA receptor. Nature.
33. Lynagh T, Lynch JW (2012) Ivermectin binding sites in human and invertebrate Cys-loop receptors. Trends Pharmacol Sci 33: 432–441. doi: 10.1016/j.tips.2012.05.002 PMID: 22677714
34. Lynagh T, Lynch JW (2012) Molecular mechanisms of Cys-loop ion channel receptor modulation by ivermectin. Frontiers in molecular neuroscience 5: 60. doi: 10.3389/fnmol.2012.00060 PMID: 22586367
35. Hibbs RE, Gouaux E (2011) Principles of activation and permeation in an anion-selective Cys-loop receptor. Nature 474: 54–60. doi: 10.1038/nature10139 PMID: 21572436
36. Krusek J, Zemkova H (1994) Effect of ivermectin on gamma-aminobutyric acid-induced chloride currents in mouse hippocampal embryonic neurones. Eur J Pharmacol 259: 121–128. PMID: 7957605
37. Dawson GR, Wafford KA, Smith A, Marshall GR, Bayley PJ, Schaeffer JM et al. (2000) Anticonvulsant and adverse effects of avermectin analogs in mice are mediated through the gamma-aminobutyric acid (A) receptor. J Pharmacol Exp Ther 295: 1051–1060. PMID: 11082440
38. Belelli D, Casula A, Ling A, Lambert JJ (2002) The influence of subunit composition on the interaction of neurosteroids with $GABA_A$ receptors. Neuropharmacology 43: 651–661. PMID: 12367610
39. Arunlakshana O, Schild HO (1959) Some quantitative uses of drug antagonists. British journal of pharmacology and chemotherapy 14: 48–58. PMID: 13651579
40. Adelsberger H, Lepier A, Dudel J (2000) Activation of rat recombinant $\alpha_1$ $\beta_2$ γ 2S $GABA_A$ receptor by the insecticide ivermectin. European journal of pharmacology 394: 163–170. PMID: 10771281
41. Colquhoun D (2007) Why the Schild method is better than Schild realised. Trends in pharmacological sciences 28: 608–614. PMID: 18023486
42. Morreale de Escobar G, Calvo R, Escobar del Rey F, Obregón MJ (1994) Thyroid hormones in tissues from fetal and adult rats. Endocrinology 134: 2410–2415. PMID: 8194467
43. Obregon MJ, MORREALE DE ESCOBAR G, ESCOBAR DEL REY F (1978) Concentrations of Triiodo-L-Thyronine in the Plasma and Tissues of Normal Rats, as Determined by Radioimmunoassay: Comparison with Results Obtained by an Isotopic Equilibrium Technique*. Endocrinology 103: 2145–2153. PMID: 748038
44. Dratman MB, Futaesaku Y, Crutchfield FL, Berman N, Payne B, Sar M et al. (1982) Iodine-125-labeled triiodothyronine in rat brain: evidence for localization in discrete neural systems. Science 215: 309–312. PMID: 7053582
45. Dratman MB, Crutchfield FL, Axelrod J, W CR, N T (1976) Localization of triiodothyronine in nerve ending fractions of rat brain. Proceedings of the National Academy of the Sciences United States of America 73: 941–944.
46. Dratman MB, Crutchfield FL (1978) Synaptosomal [125I]triiodothyronine after intravenous [125I]thyroxine. Am J Physiol 235: E638–47. PMID: 736123
47. Mason GA, Walker CH, Prange AJ (1993) L-triiodothyronine: is this peripheral hormone a central neurotransmitter? Neuropsychopharmacology 8: 253–258. PMID: 8099484

48. Sarkar PK, Ray AK (1998) Specific binding of L-triiodothyronine modulates Na(+)-K(+)-ATPase activity in adult rat cerebrocortical synaptosomes. Neuroreport 9: 1149–1152. PMID: 9601684

49. Shen W, Mennerick S, Zorumski EC, Covey DF, Zorumski CF (1999) Pregnenolone sulfate and dehydroepiandrosterone sulfate inhibit GABA-gated chloride currents in Xenopus oocytes expressing picrotoxin-insensitive GABAA receptors. Neuropharmacology 38: 267–271. PMID: 10218867

50. Stewart DS, Pierce DW, Hotta M, Stern AT, Forman SA (2014) Mutations at beta N265 in γ-aminobutyric acid type A receptors alter both binding affinity and efficacy of potent anesthetics. PLoS One 9: e111470. doi: 10.1371/journal.pone.0111470 PMID: 25347186

51. Hénin J, Salari R, Murlidaran S, Brannigan G (2014) A Predicted Binding Site for Cholesterol on the $GABA_A$ Receptor. Biophysical journal 106: 1938–1949. doi: 10.1016/j.bpj.2014.03.024 PMID: 24806926

52. Yoluk Ö, Brömstrup T, Bertaccini EJ, Trudell JR, Lindahl E (2013) Stabilization of the GluCl Ligand-Gated Ion Channel in the Presence and Absence of Ivermectin. Biophysical journal 105: 640–647. doi: 10.1016/j.bpj.2013.06.037 PMID: 23931312

53. Althoff T, Hibbs RE, Banerjee S, Gouaux E (2014) X-ray structures of GluCl in apo states reveal a gating mechanism of Cys-loop receptors. Nature 512: 333–337. doi: 10.1038/nature13669 PMID: 25143115

54. Eswar N, Webb B, Marti-Renom MA, Madhusudhan MS, Eramian D, Min-yi Shen M et al. (2007) Comparative protein structure modeling using Modeller. Current Protocols in Protein Science 2: 1–31.

55. Baumann SW, Baur R, Sigel E (2002) Forced subunit assembly in alpha1beta2gamma2 GABAA receptors. Insight into the absolute arrangement. J Biol Chem 277: 46020–46025. PMID: 12324466

56. Morris GM, Huey R, Lindstrom W, Sanner MF, Belew RK, Goodsell DS et al. (2009) AutoDock4 and AutoDockTools4: Automated docking with selective receptor flexibility. Journal of computational chemistry 30: 2785–2791. doi: 10.1002/jcc.21256 PMID: 19399780

57. Trott O, Olson AJ (2010) AutoDock Vina: improving the speed and accuracy of docking with a new scoring function, efficient optimization, and multithreading. J Comput Chem 31: 455–461. doi: 10.1002/jcc.21334 PMID: 19499576

58. Humphrey W, Dalke A, Schulten K (1996) VMD: visual molecular dynamics. Journal of molecular graphics 14: 33–38. PMID: 8744570

59. Phillips JC, Braun R, Wang W, Gumbart J, Tajkhorshid E, Villa E et al. (2005) Scalable molecular dynamics with NAMD. Journal of Computational Chemistry 26: 1781–1802. PMID: 16222654

60. MacKerell AD, Bashford D, Bellott M, Dunbrack RL, Evanseck JD, Field MJ et al. (1998) All-atom empirical potential for molecular modeling and dynamics studies of proteins. The Journal of Physical Chemistry B 102: 3586–3616. doi: 10.1021/jp973084f PMID: 24889800

61. Klauda JB, Venable RM, Freites JA, O'Connor JW, Tobias DJ, Mondragon-Ramirez C et al. (2010) Update of the CHARMM all-atom additive force field for lipids: validation on six lipid types. The journal of physical chemistry B 114: 7830–7843. doi: 10.1021/jp101759q PMID: 20496934

62. Vanommeslaeghe K, Hatcher E, Acharya C, Kundu S, Zhong S, Shim J et al. (2010) CHARMM general force field: A force field for drug-like molecules compatible with the CHARMM all-atom additive biological force fields. Journal of Computational Chemistry 31: 671–690. doi: 10.1002/jcc.21367 PMID: 19575467

63. Available: https://www.paramchem.org/ via the Internet. Accessed x.
